# Portable Multispectral Fluorometer with Embedded Machine Learning for Chlorophyll-a Estimation in Acetone-Extracted Samples

**DOI:** 10.3390/s26134086

**Published:** 2026-06-27

**Authors:** Armando Daniel Blanco-Jáquez, María Teresa Alarcón-Herrera, Luz I. Valenzuela-García, Ana Elizabeth Marín-Celestino, Samuel Villarreal-Rodríguez, Víctor M. Ayala-García, Finlandia Barbosa-Moreno, Diego Armando Martínez-Cruz

**Affiliations:** 1 Departamento de Ingeniería Sustentable, SECIHTI—Centro de Investigación en Materiales Avanzados, Calle CIMAV 110, Ejido Arroyo Seco, Durango 34147, Mexico; armando.blanco@cimav.edu.mx (A.D.B.-J.); teresa.alarcon@cimav.edu.mx (M.T.A.-H.); luz.valenzuela@cimav.edu.mx (L.I.V.-G.); samuel.villarreal@cimav.edu.mx (S.V.-R.); 2 División de Geociencias Aplicadas, SECIHTI—Instituto Potosino de Investigación Científica y Tecnológica, A.C., Camino a la Presa San José 2055, Col. Lomas 4ta Sección, San Luis Potosi 78216, Mexico; ana.marin@ipicyt.edu.mx; 3 Faculty of Chemical Sciences, Juarez University of Durango State, Durango 34120, Mexico; victor.ayala@ujed.mx; 4 Departamento de Ingeniería y Gestión Forestal y Ambiental, ETSI Montes, Universidad Politécnica de Madrid, 28040 Madrid, Spain; finlandia.barbosa@alumnos.upm

**Keywords:** chlorophyll-a, multispectral fluorescence, portable fluorometer, embedded machine learning, TinyML, ESP32-S3, acetone extraction, water quality monitoring

## Abstract

**Highlights:**

**What are the main findings?**

- A portable multispectral fluorometer was developed for chlorophyll-a estimation in acetone-extracted samples using embedded machine learning.
- The system showed promising validation performance, repeatability, agreement with the reference method, and close consistency between Python and ESP32 inference.

**What are the implications of the main findings?**

- Embedded inference enables direct chlorophyll-a estimation on the device, reducing dependence on external processing hardware after sample preparation.
- The proposed system provides a portable alternative for prepared-sample analysis when access to conventional spectrophotometric or benchtop fluorometric equipment is limited.

**Abstract:**

Chlorophyll-a (Chl-a) is commonly used as an indicator of phytoplankton biomass and water quality, but its determination usually depends on laboratory-based methods and specialized equipment. In this work, a portable multispectral fluorometer was developed and evaluated for Chl-a estimation in 90% acetone extracts prepared from water samples. The system integrates a nominal 420–425 nm fluorescence excitation source, 90° detection geometry, two multispectral sensors (AS7341 and AS7343), an ESP32-S3 microcontroller, and an Android application for device control and result visualization. A dataset of 76 samples was used for model development, including regression-model benchmarking, model selection, and internal testing. A Random Forest model selected for embedded implementation was exported to C++ for on-device inference. Validation using 15 independent samples yielded *R*^2^ = 0.979, root mean square error (RMSE) = 35.41 µg/L, mean absolute error (MAE) = 28.91 µg/L, and mean absolute percentage error (MAPE) = 6.14%. Agreement analysis showed a positive bias of 22.00 µg/L, while repeatability tests showed a maximum coefficient of variation (CV) of 3.29%. Embedded inference was highly consistent with Python predictions (*R*^2^ = 0.997). These results suggest that the system is a promising portable alternative for Chl-a estimation in prepared acetone extracts under the evaluated conditions.

## 1. Introduction

Chlorophyll-a (Chl-a) is widely used as an indicator of phytoplankton biomass [1,2]. As a primary photosynthetic pigment, its concentration is commonly used to assess water quality and monitor eutrophication processes in aquatic systems [3]. Elevated Chl-a levels are also associated with algal blooms, which can affect ecosystem health and water quality [2]. For this reason, accurate and reliable measurement of Chl-a is important for environmental monitoring and water resource management [1].

Traditionally, Chl-a concentration has been determined using laboratory-based techniques such as spectrophotometry, fluorometry, and high-performance liquid chromatography (HPLC) [1,4]. These methods are widely accepted and provide reliable measurements of chlorophyll concentration [5,6,7]. However, they require sample preparation steps, including pigment extraction using organic solvents such as acetone or ethanol [4]. Besides sample preparation, the analysis also relies on specialized benchtop instrumentation and controlled conditions, which can limit flexibility and increase operational cost [8]. These approaches are suitable for laboratory environments, but less practical when a simplified workflow or more accessible instrumentation is required [9]. Therefore, portable instrumentation for prepared extracts remains relevant because it can reduce dependence on centralized laboratories or benchtop equipment after the extraction step has been completed. This type of system can provide a simplified measurement option for prepared-sample analysis in small laboratories, field stations, or decentralized monitoring settings where access to spectrophotometric, fluorometric, or chromatographic instrumentation may be limited. In this context, alternative approaches have been developed to reduce dependence on conventional laboratory equipment while maintaining reliable measurements [10].

Recent studies have explored optical sensors and portable fluorometric systems to improve the accessibility of Chl-a estimation [10,11]. In particular, low-cost multispectral approaches based on multi-wavelength LED arrays have been proposed to increase flexibility and capture more detailed optical responses [11]. In parallel, data-driven machine learning models have been used for Chl-a estimation from spectral and multispectral information, showing their ability to model complex relationships between optical features and pigment concentration [12,13].

Despite these advances, many portable or low-cost Chl-a measurement systems still separate optical acquisition from data processing, relying on external software environments, post-analysis, or additional computational resources [14,15]. In these cases, the device functions mainly as an acquisition unit rather than as a standalone measurement device. Although multispectral sensing can provide richer optical information than single-channel fluorescence detection, its integration with embedded machine learning for direct Chl-a estimation remains limited in prepared-sample workflows [10,16]. This limits their ability to operate as standalone instruments capable of providing local estimates without additional computational resources [17]. In addition, embedded machine learning (TinyML) has become an important approach for implementing predictive models in resource-constrained devices [18,19], but its use for on-device inference in portable fluorometric Chl-a analysis has not yet been widely explored.

Although portable optical setups [10,11,14,15] and embedded deployment strategies [17,18,19] have already been reported in different optical sensing applications, the contribution of the present study is positioned at the system-integration level rather than at the individual component level. Specifically, this work proposes and evaluates a portable multispectral fluorometer for Chl-a estimation in acetone-extracted water samples.

The system integrates fluorescence excitation, dual multispectral optical acquisition, blank correction, embedded machine learning inference, local data storage, and wireless visualization through an Android application within an integrated workflow. A dataset of extracted samples was used for model development and validation, and the embedded implementation was compared against the original Python model to evaluate numerical consistency after deployment.

The main contributions of this work are as follows: (1) the design and implementation of an integrated portable multispectral fluorescence system for Chl-a estimation in acetone-extracted samples; (2) the deployment of an embedded-compatible regression model for direct on-device inference on an ESP32-S3 platform; and (3) the experimental evaluation of the integrated workflow, including model performance, agreement with the reference method, repeatability, drift behavior, and consistency between Python and embedded inference.

## 2. Materials and Methods

### 2.1. Methodological Overview

**Figure 1:**
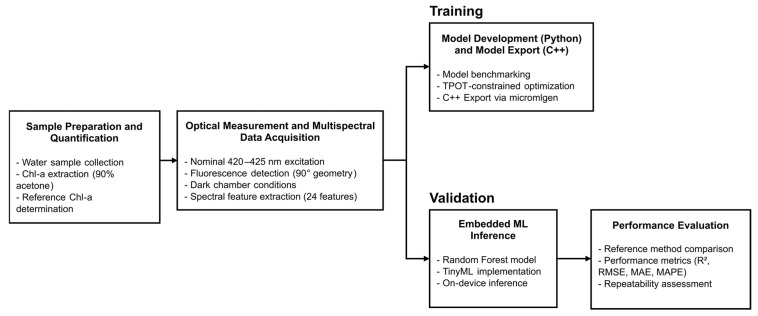
Methodological workflow of the proposed portable fluorometer. The process includes sample preparation and reference chlorophyll-a (Chl-a) determination, followed by optical measurement (nominal 420–425 nm excitation, 90° detection geometry, dark chamber conditions) and multispectral data acquisition. The workflow is then divided into model training, where machine learning models are developed and exported for embedded implementation, and validation, where new samples are analyzed using on-device inference and compared against the reference method to assess performance and repeatability.

The general methodology followed in this work is shown in Figure 1, where the process is divided into two main stages: model training and validation.

For model development, a dataset of 76 extracted samples was first generated from water samples collected in the field. The Chl-a concentration of each sample was determined using a reference method, and the same samples were then measured with the developed fluorometer. This allowed establishing the relationship between the acquired spectral response and the corresponding experimentally determined Chl-a values. Based on this dataset, several machine learning (ML) models were evaluated through a benchmarking process, from which a final model was selected. The selected model was then exported to C++ and implemented on the embedded system.

Once the model was integrated into the device, 15 independent water samples not included in the training dataset were collected, processed, and analyzed. Measurements were performed following the same sample preparation and measurement workflow used during the training stage. The signals were processed directly on the device, and the embedded model was used to estimate Chl-a concentration in μg/L. Finally, the predicted values were compared with the reference measurements to evaluate the performance of the model and the overall measurement reliability.

### 2.2. System Architecture and Measurement Principle

**Figure 2:**
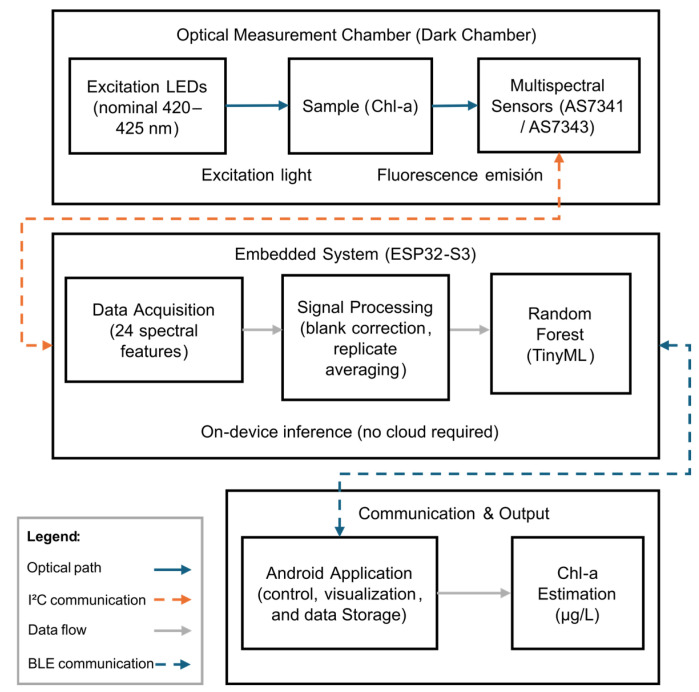
System architecture of the portable fluorometer, including the optical measurement chamber, multispectral sensing stage, and embedded processing unit (ESP32-S3). Fluorescence excitation (nominal 420–425 nm) and detection (90° geometry) are combined with multispectral data acquisition via I^2^C. The acquired signals are processed on-device and used for chlorophyll-a (Chl-a) estimation through an embedded machine learning model. Results are transmitted via BLE to a custom Android application developed for this study (version 1.0.0). Different system interactions are indicated in the figure.

The system developed in this work consists of a portable fluorometer designed to perform in vitro Chl-a measurements under controlled conditions. In general, the device integrates an optical measurement stage, a multispectral sensing configuration, and an embedded system for data acquisition and processing, as shown in Figure 2.

The optical setup was implemented inside a dark chamber in order to reduce the influence of ambient light during measurements. Fluorescence excitation was provided by a nominal 420–425 nm LED (OTdiode, Shenzhen, China), selected because its emission is close to the blue excitation wavelength commonly recommended for fluorometric determination of Chl-a extracts and lies within the excitation region used in chlorophyll fluorescence measurements [20,21]. The excitation light is directed towards the sample using a collimator (ZhonNa, Zhongshan, China), while the emitted fluorescence is detected at a 90° geometry. This configuration helps to reduce the direct impact of the excitation light on the sensors, improving the signal-to-noise ratio of the acquired measurements.

Samples are placed in a quartz cuvette (Yixing Purshee Optical Elements Co., Ltd., Yixing, China) located within the measurement chamber. The fluorescence signal is captured using two multispectral sensors (AS7341 and AS7343, ams OSRAM, Premstätten, Austria), which together provide 24 spectral channels. This dual-sensor configuration was used to obtain a wider spectral representation of the fluorescence response.

The electronic system is based on an ESP32-S3 microcontroller (Espressif Systems, Shanghai, China). This unit is responsible for managing sensor communication, data acquisition, and signal processing. Communication with the multispectral sensors is carried out through the I^2^C protocol. Once the data are acquired, basic processing is performed on-device, including blank correction and replicate averaging.

The machine learning model used in this work is based on a Random Forest algorithm. The model was trained in Python version 3.12.9 and later exported to C++ for implementation on the microcontroller as part of a TinyML-based embedded inference strategy. This allows the device to estimate Chl-a concentration directly on the system, without the need for external processing. Finally, the results are transmitted to an Android application via Bluetooth Low Energy (BLE), where they can be visualized and stored for further analysis.

### 2.3. Optical Measurement Setup

**Figure 3:**
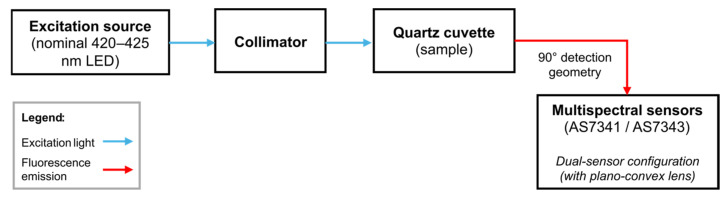
Optical setup of the portable fluorometer, including the nominal 420–425 nm excitation source, collimator, quartz cuvette, and dual multispectral sensing configuration (AS7341 and AS7343) arranged at a 90° detection geometry. A plano-convex lens was placed in front of each sensor to improve fluorescence light collection and direct the emitted signal toward the sensing area.

The optical setup used in this work is shown in Figure 3. The sample was excited using a nominal 420–425 nm LED. The emitted light from the source was guided towards the quartz cuvette using a collimator, mainly to reduce dispersion before reaching the sample.

The multispectral sensors were positioned orthogonally to the excitation path. This 90° detection geometry is commonly used in fluorescence measurements, since it limits the direct influence of excitation light on the sensors and reduces scattering effects [22].

Two multispectral sensors (AS7341 and AS7343) were used in the system, each one with its own optical path. Compact commercial multispectral sensors such as the AS7341 have previously been explored for low-cost fluorescence detection, showing their potential for miniaturized optical instrumentation [23]. This configuration allows the same sample to be measured without additional handling. In addition, combining both sensors extends the effective spectral coverage, particularly in the 600–700 nm region, where a relevant fluorescence emission band of Chl-a is expected [20,21]. Similar multi-sensor strategies have been reported to improve spectral reconstruction when using commercial spectral sensors [24].

To provide a minimal spectral description of the optical acquisition subsystem, Table 1 summarizes the nominal characteristics of the multispectral detector channels used as predictors in the regression model. The excitation source was a 1 W LED with a nominal emission range of 420–425 nm, according to the supplier specification. The spectral information reported for the AS7341 and AS7343 sensors corresponds to manufacturer-reported nominal center/peak wavelengths and typical full width at half maximum (FWHM) values. These data provide a component-level spectral description of the optical subsystem, although a complete spectroradiometric characterization of the assembled optical system was not performed.

Both multispectral sensors were operated under fixed acquisition settings during all measurements. The AS7341 and AS7343 were configured with a gain of 256× and integration parameters ATIME = 99 and ASTEP = 799. Each sensor was connected to the ESP32-S3 through an independent 400 kHz I^2^C bus. Measurements were performed using a quartz cuvette with a 1 cm optical path length.

A plano-convex lens (Rhuxyol, China) was placed in front of each sensor to improve light collection efficiency and direct the emitted fluorescence onto the sensing area, following general manufacturer recommendations. To keep all components in place and reduce variability between measurements, the system was enclosed in a 3D-printed dark chamber made from black PLA. This also reduces the effect of ambient light during the measurements.

### 2.4. Hardware Implementation

**Figure 4:**
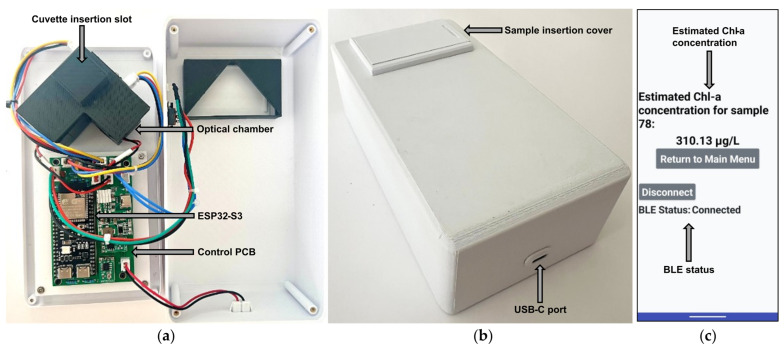
Hardware implementation of the developed portable fluorometer. (**a**) Internal view showing the ESP32-S3 microcontroller, control PCB, optical chamber, and cuvette insertion slot. (**b**) External view of the 3D-printed enclosure, including the sample insertion cover and USB-C port. (**c**) Android application interface used for device operation and visualization of the estimated chlorophyll-a (Chl-a) concentration.

The device was implemented as a portable system designed to operate using a mobile phone as the main interface. Control of the system is carried out through an Android application, which communicates with the device via Bluetooth Low Energy (BLE) (Figure 4c).

For data processing, an ESP32-S3 development module was used as the main processing unit (Figure 4a). This microcontroller was selected due to its processing and memory capabilities, which are sufficient for running the selected machine learning model, while maintaining stable communication with both the sensors and the mobile application.

The ESP32-S3 is responsible for controlling the multispectral sensors (AS7341 and AS7343) through the I^2^C bus. In addition, it manages the synchronization between each sensor and its corresponding excitation LED. The LEDs were driven using a constant current driver module (LDO6AJSA), based on the CN5711 integrated circuit, which helps to maintain stable illumination conditions during the measurements.

To ensure portability, the system was powered using a rechargeable 5000 mAh lithium battery, together with a charging controller module. All electronic components were integrated into a custom PCB, allowing a more stable and reliable operation compared to prototyping setups (Figure 4a).

The mechanical design includes a 3D-printed enclosure with a dedicated access window for the dark chamber, as well as external elements such as a power switch, a USB-C charging port, and a status LED for battery indication (Figure 4b).

The Android application provides two main functions: blank registration and sample measurement. During measurement, the user can assign an identifier to each sample, and once the process is completed, the estimated chlorophyll-a value is displayed on the screen. Both the predicted concentration and the 24 spectral features are stored locally in the ESP32 for further analysis.

### 2.5. Data Acquisition, Signal Processing and Dataset Generation

For dataset generation and validation, a total of 91 extracted samples were analyzed. Of these, 76 samples were used for model development, including benchmarking, model selection, training, and internal testing, while 15 independent samples were used for independent validation. The model-development dataset was acquired using an earlier prototype version of the device, whereas the independent validation dataset was obtained using the final hardware version. Therefore, the independent validation also served as a preliminary assessment of model transfer between hardware versions, but not as a complete final-hardware recalibration. Water samples were collected from different freshwater bodies located near Durango, Mexico, to obtain extracts covering a broad range of Chl-a concentrations.

A volume of 1 L was collected for each sample in opaque plastic containers to minimize light exposure during transport. Each sample was labeled with a unique identifier and kept in a cooler with ice until laboratory processing. Chlorophyll-a extraction and reference quantification were performed based on EPA Method 446.0 for the in vitro spectrophotometric determination of chlorophyll pigments [6], with practical adaptations to improve filtration efficiency.

Briefly, each sample was first passed through a 47 mm glass-fiber prefilter with a 1.6 µm pore size (JVLAB, Foshan, China) to remove larger suspended particles and reduce premature clogging, followed by filtration through a 47 mm glass-fiber filter with a 0.7 µm pore size (JVLAB, Foshan, China), as specified in the reference method. After filtration, the filters containing the retained pigments were stored at −80 °C until extraction.

For pigment extraction, the filters were macerated with 90% acetone, and the final extraction volume was adjusted to 20 mL with 90% acetone. The extracts were maintained under dark conditions for 24 h at 4 °C to promote pigment extraction. After extraction, the samples were centrifuged at 4500× *g* for 10 min at 4 °C using a temperature-controlled Sorvall ST 40R centrifuge (Thermo Fisher Scientific, Waltham, MA, USA), and the supernatant was recovered for spectrophotometric analysis.

For the spectrophotometric reference measurement, 3 mL of the supernatant was transferred to a quartz cuvette with a 1 cm optical path length and analyzed using a DR 5000™ UV-Vis spectrophotometer (Hach Company, Loveland, CO, USA). A solvent blank consisting of 90% acetone was measured to account for the absorbance contribution of the extraction solvent. Absorbance was then measured at 750, 664, 647, and 630 nm. The absorbance at 750 nm was used for turbidity/background correction of the pigment absorbance values. Chlorophyll-a concentration in the acetone extract was calculated using the trichromatic equation of Jeffrey and Humphrey:

(1) $$C_{E,a}=11.85\left(Abs\;664\right)-1.54\left(Abs\;647\right)-0.08\left(Abs\;630\right)$$

where $C_{E,a}$ is the chlorophyll-a concentration (mg/L) in the extraction solution analyzed, and Abs 664, Abs 647, and Abs 630 correspond to the corrected absorbance values at each wavelength. The chlorophyll-a concentration with respect to the original water sample was then calculated by accounting for the extraction volume, dilution factor, filtered sample volume, and cuvette optical path length:

(2) $$C_{s}=\;\frac{C_{E,a}\;\times \;extract\;volume\;\left(L\right)\;\times \;DF}{sample\;volume\;\left(L\right)\;\times \;cell\;length\;\left(cm\right)}$$

where $C_{s}$ is the chlorophyll-a concentration (mg/L) in the original water sample, $C_{E,a}$ is the concentration (mg/L) of pigment in extract measured in the cuvette, extract volume is the volume (L) of extract, *DF* is the dilution factor, sample volume is the volume (L) of the original water sample that was filtered, and cell length is the optical path length (cm) of cuvette used. The calculated $C_{s}$ values were then converted from mg/L to µg/L for reporting, model development, and validation. The resulting spectrophotometric values were used as reference concentrations for model development and validation.

Before each measurement with the developed device, a blank was registered in the system in order to account for the signal contribution of the solvent, in this case 90% acetone. This value was later used for baseline correction of all measurements.

For each sample, 3 mL of the extracted solution were placed in the quartz cuvette. The cuvette was then inserted into the dark chamber, and the measurement was performed.

Each measurement was automatically repeated three times for each multispectral sensor. The corresponding blank value was subtracted from each reading, and the final spectral response was calculated as the average of the three measurements, resulting in a single spectral vector per extracted sample. These values were stored in the ESP32 for later processing.

All dataset partitions were performed at the independent-sample level, not at the individual instrumental-reading level. Thus, repeated readings from the same sample were never assigned to different subsets, preventing methodological leakage between training, internal testing, and independent validation.

The final model-development dataset consisted of 76 samples, each identified by a unique numerical identifier. For each sample, a total of 24 spectral features were obtained from the sensors, together with the corresponding Chl-a concentration (µg/L) determined by the reference method.

### 2.6. Machine Learning Model Development and Embedded Implementation

The purpose of the model-development workflow was to estimate Chl-a concentration from the 24 spectral features obtained from the multispectral sensors while producing a model suitable for microcontroller deployment. In this study, the machine-learning workflow was designed as an embedded-compatible model-selection and deployment strategy. The emphasis was placed on comparing regression models, selecting a predictive model suitable for ESP32-S3 implementation, and verifying that the exported C++ model preserved the numerical behavior of the original Python implementation. To evaluate the suitability of different approaches, a benchmarking analysis was performed using five regression models.

The evaluated models included two linear models (Ridge and Lasso) [25,26], a kernel-based model (Support Vector Regressor) [27], and two ensemble methods based on decision trees (Random Forest and Gradient Boosting Machine) [28,29]. All machine learning analyses were performed in Python using scikit-learn (version 1.6.1). The 24 spectral features obtained from both multispectral sensors were used as predictors for all evaluated models. For the final embedded model, the 24 channels were retained to preserve the complete dual-sensor multispectral response and to maintain a fixed input structure for embedded deployment, rather than introducing an additional custom feature-screening step. Each model was evaluated using 5-fold cross-validation in order to compare their performance under the same conditions [25,30]. A fixed random state of 42 was used throughout the model-development workflow to improve reproducibility.

Because fluorescence responses can exhibit non-linear behavior, particularly at higher concentrations, the final model selection also considered the ability to capture potential non-linear relationships while remaining suitable for embedded deployment [12,13,22].

Considering these requirements, an AutoML approach was then used to guide the final model selection [31]. The TPOT library (version 0.12.2) in Python was used for this purpose [32], applying a constrained optimization in which the search space was intentionally limited to models compatible with C++ export using micromlgen (version 1.1.28) [33]. In this case, the search was restricted to DecisionTreeRegressor and RandomForestRegressor models.

For the TPOT optimization, the 76-sample model-development dataset was divided into training and internal holdout subsets using an 80:20 split with a fixed random state of 42. The optimization was carried out using 5 generations, a population size of 50, 5-fold cross-validation, and a single-regressor template to avoid complex pipelines that could not be directly implemented on the microcontroller. Four scoring criteria were evaluated separately: R^2^, mean absolute error, root mean square error, and mean absolute percentage error, using the corresponding negative scoring functions when required by scikit-learn.

These methodological choices also helped reduce, but not eliminate, overfitting risk associated with the limited sample-to-feature ratio. Specifically, the workflow included comparison of models with different complexity levels, internal testing, cross-validation during model selection, a constrained AutoML search space limited to embedded-compatible regressors, and exclusion of complex stacked or multi-step pipelines. The selected model was subsequently evaluated using an independent validation dataset that was not used during model training, model selection, benchmarking, or internal testing. Nevertheless, the limited sample-to-feature ratio remained a constraint on model generalizability and is therefore acknowledged as a limitation.

The final selected model was a RandomForestRegressor with 100 trees, max_depth = None, min_samples_split = 2, min_samples_leaf = 2, and random_state = 42. This model achieved performance comparable to the best benchmarked models while maintaining a structure suitable for deployment on the microcontroller.

Once the model was selected, the fitted model was exported to C++ using the micromlgen library [33]. The generated code was integrated into the ESP32-S3 firmware, allowing on-device inference. During operation, the 24 spectral features are organized in the same order used during training and provided as input to the embedded model, which returns the estimated Chl-a concentration in µg/L.

### 2.7. Model Validation and Performance Evaluation

During model development and final validation, performance was assessed using the following regression metrics: coefficient of determination (*R*^2^), root mean square error (RMSE), mean absolute error (MAE), and mean absolute percentage error (MAPE) [25,34].

In addition to the regression metrics, the residual predictive deviation (RPD) was calculated as the ratio between the standard deviation of the reference values and the RMSE. An approximate residual-based expanded uncertainty (U) was also estimated using a coverage factor of *k* = 2, based on the standard deviation of the residuals.

For model validation, a set of 15 independent water samples, collected after the model development dataset, was used. These samples were not included in model training, benchmarking, model selection, or internal testing. All samples were processed and measured following the same procedure described previously, so that the predicted values could be directly compared with the reference measurements.

To evaluate measurement consistency, a repeatability analysis was also performed. For this purpose, three samples were selected to represent low, medium, and high Chl-a concentrations within the independently validated interval. These samples were classified as low, medium, and high concentration. Each one was measured 10 times under non-consecutive conditions.

This procedure was adopted because consecutive measurements on the same sample showed a progressive decrease in the measured signal, which is consistent with the photobleaching effect [22]. To reduce this effect, the measurements were carried out in cycles, starting with the low concentration sample, followed by the medium and high concentration samples, and then repeating the sequence until the 10 measurements were completed for each level.

For the repeatability analysis, the mean value, standard deviation (SD), and coefficient of variation (CV) were calculated for each sample. In addition, the term *r* = 2.8·SD was used as an approximate repeatability-limit indicator, consistent with the conventional repeatability-limit expression for repeated measurements under normal-error assumptions [35].

A drift analysis was also carried out by fitting a linear model to the repeated measurements of each sample. From this, the slope, the estimated total drift, the coefficient of determination (*R*^2^), and the associated *p*-value were obtained.

An additional analysis by concentration ranges was performed in order to identify possible differences in system performance across the measurement range. Agreement between the proposed system and the reference method was further evaluated using Bland–Altman analysis [36], residual plots, scatter plots, and bias as a function of concentration.

Finally, the embedded implementation was verified by comparing the predictions obtained directly on the ESP32-S3 with those generated by the original model before conversion, using the same multispectral measurements as input.

## 3. Results

### 3.1. Dataset Characteristics

In total, 76 water samples were used during the model-development stage. The Chl-a concentration ranged from 115.42 to 1809.13 µg/L, with an average value of 600.42 µg/L. Concentrations across the whole dataset were widely dispersed, as reflected by a coefficient of variation of 81.6%. A more detailed description is given in Table 2.

**Figure 5:**
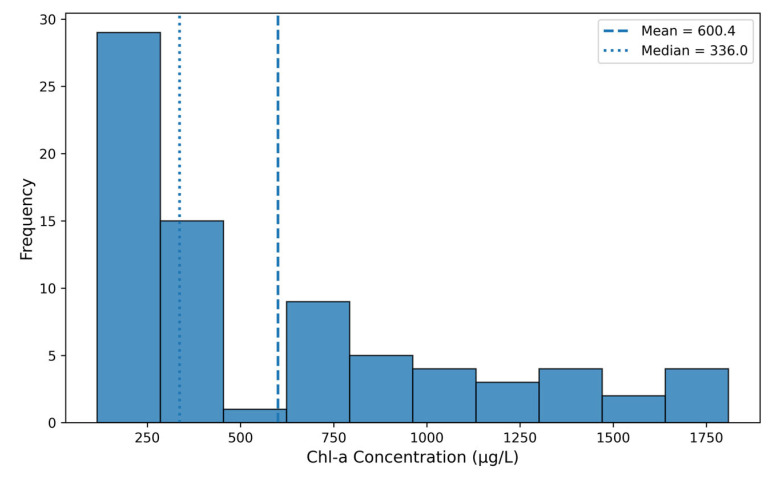
Distribution of chlorophyll-a (Chl-a) concentrations in the model-development dataset. Most samples are concentrated in the lower range, while higher concentrations appear less frequently, resulting in a positively skewed distribution.

The distribution of the samples is presented in Figure 5. Most of the samples fall within the lower concentration range, mainly below 500 µg/L, while higher values appear only in a few cases. This can also be observed when comparing the mean and median values (600.4 and 336.0 µg/L), which indicate a positively skewed distribution, characterized by a higher concentration of samples at lower values and a tail extending towards higher concentrations.

This kind of distribution is expected when working with environmental water samples, where lower concentrations are more common, while higher values tend to appear less frequently [2]. Even so, the model-development dataset still spanned a wide concentration range, which supported model training and internal evaluation across different concentration levels.

As an additional quality-control step, the raw digital outputs of the multispectral channels were inspected to identify possible saturation. The maximum observed channel value was 54,222 digital counts, corresponding to approximately 82.7% of the 16-bit upper digital limit of 65,535 counts. Therefore, no digital saturation was observed in the channels used for model development and independent validation.

### 3.2. Model Benchmarking and Selection

The benchmarking results for the evaluated models are shown in Table 3. In general, all models showed similar performance, with the exception of SVR, which showed the lowest performance across the evaluated metrics. The best results were obtained with Ridge and Lasso, followed closely by the ensemble models.

Although Ridge and Lasso showed the best numerical performance during benchmarking, model selection also considered practical implementation on the embedded platform. In this context, the selected model had to be compatible with C++ export and stable enough for on-device inference under the prepared-sample workflow.

The restricted TPOT optimization selected a Random Forest model when *R*^2^ and RMSE were used as scoring criteria. In both cases, the best pipeline corresponded to a RandomForestRegressor with max_depth = None, min_samples_leaf = 2, min_samples_split = 2, and n_estimators = 100. This model obtained *R*^2^ = 0.991, RMSE = 38.99 µg/L, MAE = 24.38 µg/L, and MAPE = 4.06% on the internal holdout set used during model development.

Under these conditions, the final Random Forest model was selected because it provided performance close to the best benchmarked models while remaining directly compatible with the embedded implementation. In addition, its non-linear structure provides the ability to capture potential non-linearities in the fluorescence response, including effects such as self-absorption, quenching, and inner-filter effects at higher concentrations [22,37].

### 3.3. Validation with Independent Samples

The independent validation dataset included a total of 15 samples, with Chl-a concentrations ranging from 203.74 to 934.94 µg/L. Therefore, the validation results should be interpreted within this independently validated concentration interval. Although the model-development dataset covered a wider range of 115.42 to 1809.13 µg/L, performance at the lower and upper extremes, particularly above 934.94 µg/L, was not independently confirmed in this study. The evaluation results are summarized in Table 4, where n=15 corresponds to the independent validation samples used to evaluate the predictive performance of the final model.

In general, the model showed promising predictive performance relative to the reference measurements. An *R*^2^ value of 0.979 was obtained, together with an RMSE of 35.41 µg/L. The MAE and MAPE values (28.91 µg/L and 6.14%, respectively) also remained relatively low for the independently validated samples. Besides this, the RPD value of 7.20 suggests favorable predictive performance according to commonly used spectroscopic model evaluation criteria, although this interpretation should be considered together with the limited size of the independent validation set [38].

A small positive bias of 22.00 µg/L was observed, indicating a slight tendency to overestimate the concentration. Even so, this deviation remains small when compared with the variability observed in the independent validation dataset. This bias may be partially associated with systematic differences in acquisition conditions between the earlier prototype used for model development and the final hardware used for validation, particularly related to excitation LED control. Therefore, the observed bias should be interpreted not only as a prediction residual, but also as a possible hardware-transfer effect.

The results obtained using local inference on the ESP32 were consistent with the Python-model predictions for the independent validation samples, indicating that the embedded implementation preserved the numerical behavior of the original model.

**Figure 6:**
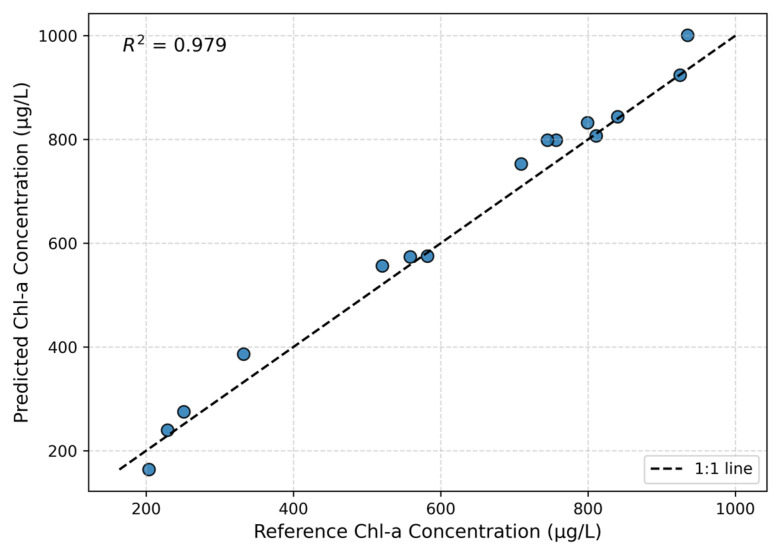
Comparison between predicted and reference chlorophyll-a (Chl-a) concentrations for the independent validation dataset. Blue circles represent individual independent validation samples, and the dashed line represents the 1:1 relationship.

Figure 6 shows the relationship between predicted and reference values. Most of the points remain close to the 1:1 line, and no clear outliers can be observed. Under these conditions, the dispersion remains relatively uniform within the independently validated concentration interval.

### 3.4. Agreement Analysis with the Reference Method

The agreement between the proposed system and the reference method was further analyzed using Bland–Altman and residual plots. These representations help to visualize how the prediction error behaves across the independently validated concentration interval.

**Figure 7:**
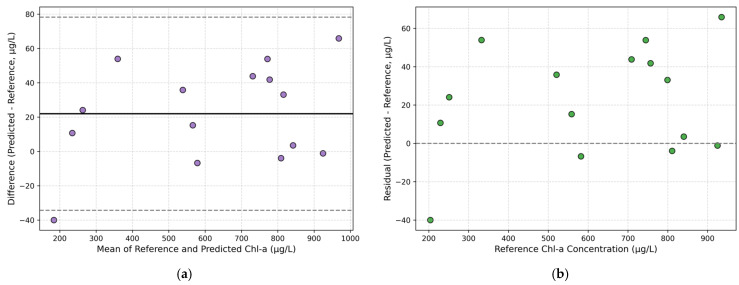
(**a**) Bland–Altman plot comparing the chlorophyll-a (Chl-a) concentrations predicted by the developed system and those obtained with the reference method. Purple circles represent individual independent validation samples. The solid line represents the mean bias (22.0 µg/L), while the dashed lines indicate the 95% limits of agreement (−34.3 to 78.3 µg/L). (**b**) Residuals of the predicted Chl-a concentrations with respect to the reference values across the independently validated concentration interval. Green circles represent individual independent validation samples, and the dashed line indicates zero residual.

The Bland–Altman plot is shown in Figure 7a. A mean bias of 22.0 µg/L was observed, consistent with the positive bias previously reported in the validation metrics. Based on the residual standard deviation, the approximate 95% limits of agreement were −34.3 to 78.3 µg/L. Most of the points remain within these limits, and no extreme values are observed. This indicates that the differences between predicted and reference values were limited for the evaluated samples.

The difference between the two methods does not show a clear dependence on Chl-a concentration, suggesting that no strong proportional bias is present within the independently validated concentration interval.

In general, the residuals remain relatively stable, although a tendency toward positive values is observed, as shown in Figure 7b. This behavior is consistent with the previously observed overestimation.

Under these conditions, the model showed a relatively stable response for the evaluated samples. Some dispersion is observed at higher concentrations, but no clear pattern of systematic deviation is evident.

### 3.5. Repeatability and Drift Analysis

The results of the repeatability analysis are summarized in Table 5. The sample size reported in this analysis corresponds to 10 repeated instrumental measurements for each selected concentration level. In this context, the repeatability-limit indicator (*r* = 2.8·SD) provides an approximate estimate of the expected variability between repeated measurements under the adopted measurement conditions. In this case, it ranges from 2.55 µg/L (low concentration) to 90.43 µg/L (high concentration).

In general, low variability between repeated measurements was observed, with a maximum coefficient of variation of 3.29%. These low CV values indicate good short-term repeatability of the system, particularly at low and medium concentrations. As expected, variability increases with concentration, as shown by the standard deviation and CV values.

**Figure 8:**
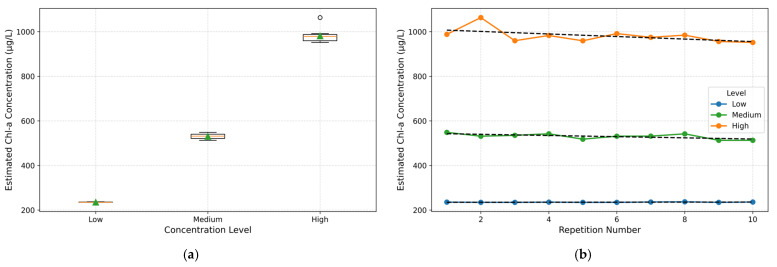
(**a**) Box plot of repeated measurements for three chlorophyll-a (Chl-a) concentration levels, showing the increase in variability with concentration. The horizontal line inside each box represents the median, and the green triangle represents the mean. (**b**) Sequential measurements for three Chl-a concentrations, showing the drift behavior across repetitions and the tendency toward signal decrease at higher concentrations. Dashed lines represent the fitted linear trend for each concentration level.

This trend is also visible in the box plot shown in Figure 8a. Higher concentrations show greater dispersion than lower concentrations, although the measurements remain within a relatively narrow range. Only a few outliers are present.

Figure 8b shows the behavior across repetitions. A slight increase in dispersion appears at medium and high concentrations. Besides this, a small negative trend is observed in these cases, consistent with the negative slopes reported in Table 5, around −5 µg/L per measurement at the highest concentration. The relatively low *R*^2^ values indicate that this drift trend accounts for only a portion of the variability observed across repeated measurements.

A statistically detectable negative trend was observed only at the medium concentration level (*p* = 0.04), whereas no statistically significant drift was observed at the low and high concentration levels. However, this result should be interpreted cautiously due to the small number of repeated measurements and because the drift analysis was performed separately for three concentration levels.

This effect was also observed during the experimental procedure. When measurements were performed consecutively on the same sample, a more pronounced decrease in the estimated concentration was detected. For this reason, a sequential measurement approach was used, alternating between samples to reduce this effect. Despite this, a small drift tendency remains, particularly at medium and high concentrations.

Under these conditions, the system showed good short-term repeatability across the evaluated concentrations, with limited variability and a small negative drift tendency that should be further evaluated with a larger number of repeated measurements.

### 3.6. Embedded Implementation Consistency

The results of the analysis evaluating the consistency between the Python model and its embedded implementation are summarized in Table 6. In general, the inference performed on the ESP32 shows a close agreement with the values obtained from the original Python model.

An MAE of 10.75 µg/L and an RMSE of 14.81 µg/L were obtained, indicating a relatively small difference between the two implementations, considering the independently validated concentration interval. The bias remains close to zero (−0.07 µg/L), suggesting no systematic deviation between the two approaches. Besides this, the maximum absolute difference was 35.63 µg/L.

**Figure 9:**
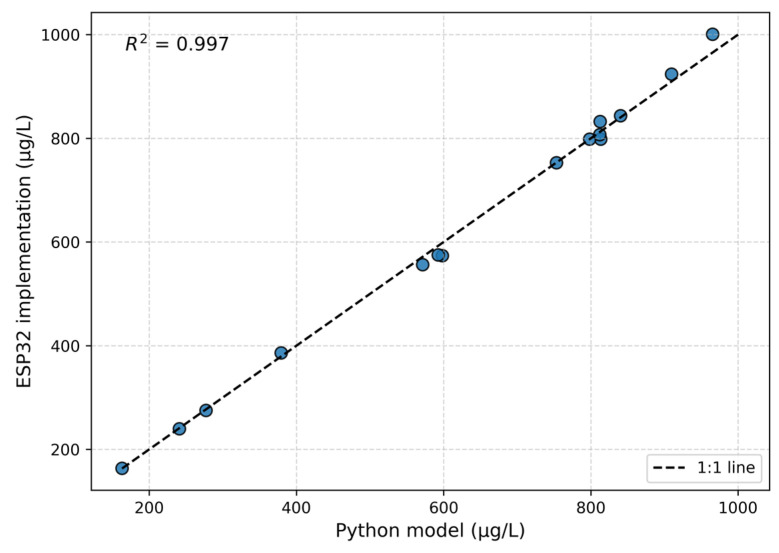
Comparison between the chlorophyll-a (Chl-a) concentrations predicted by the Python model and those obtained from the embedded implementation on the ESP32. Blue circles represent individual samples used for the embedded-implementation consistency assessment, and the dashed line indicates the 1:1 relationship.

Nevertheless, the overall numerical consistency between the two implementations was high, as reflected by an *R*^2^ value of 0.997. This relationship is also shown in Figure 9.

Under these conditions, the embedded implementation preserved the numerical behavior of the original Python model, with only minor differences between implementations.

## 4. Discussion

### 4.1. Overall Performance and Model Behavior

In general, the proposed system showed promising performance for the independent validation samples, which covered a concentration interval of 203.74–934.94 µg/L. The validation results indicate that the model was able to capture the relationship between the multispectral response and Chl-a concentration, with prediction errors that were relatively small compared with the variability observed in the validation dataset.

However, the model-development dataset was not uniformly distributed, with a higher density of samples at lower concentrations. In this context, the model tended to perform more consistently within this range, while slightly higher dispersion was observed toward the upper end of the independently validated interval. This behavior is expected, since fluorescence-based measurements of chlorophyll are known to exhibit non-linear effects at higher concentrations, including self-absorption, quenching, and inner-filter effects, which can affect the proportionality between fluorescence intensity and concentration [22,37].

### 4.2. Comparison with Existing Approaches

Direct comparison with previous studies is not straightforward because reported Chl-a sensing approaches differ in sample type, optical configuration, calibration strategy, and processing workflow. Some systems are designed for in situ or raw-water measurements, whereas others are intended for extracted samples or laboratory-assisted analysis. In addition, some approaches rely on commercial fluorometers, remote sensing data, or external software-based processing, which makes direct metric-by-metric comparison difficult.

Even with these differences, the performance reported in this work is consistent with previous studies that evaluated fluorescence-based methods for Chl-a estimation. Handheld and portable fluorometers have been widely used for rapid fluorescence-based estimation of Chl-a, although their accuracy depends strongly on calibration conditions and sample characteristics [8]. In addition, low-cost optical systems based on LED excitation and fluorescence detection have been proposed as alternatives to laboratory methods, showing that portable instrumentation can achieve reliable measurements under controlled conditions [11].

In this context, the proposed system differs from conventional fluorometers by integrating multispectral acquisition, blank correction, and machine learning-based estimation within a single portable platform. While data-driven approaches have also been reported for Chl-a estimation, many of them rely on external processing or post-analysis [12]. In contrast, the implementation presented here allows inference to be performed directly on the device, reducing dependence on external processing and enabling immediate estimates after sample preparation.

### 4.3. Repeatability and Measurement Stability

During the repeatability analysis, a small negative trend was observed in the repeated measurements, particularly at medium and high concentrations. This behavior is consistent with photobleaching, in which repeated exposure can produce a decrease in fluorescence over time. Similar effects have been reported in fluorescence measurements, where signal stability can be affected by repeated exposure and sample conditions [22].

The relatively low *R*^2^ values obtained in the drift analysis indicate that this trend explains only part of the observed variability, suggesting the presence of other sources of variation. Switching from consecutive to sequential measurements helped reduce this effect, although it was not eliminated.

In addition, a small positive bias was identified during validation and confirmed by residual analysis. This may be related to differences between the conditions used to obtain the model-development and validation datasets. The model-development data were obtained using an earlier prototype version of the device, while the validation measurements were performed with the final hardware version. During this transition, adjustments in the excitation LED control were introduced, which can affect the intensity and stability of the excitation signal. Under these conditions, a consistent positive shift in the fluorescence response can occur. This behavior is consistent with previous studies reporting that chlorophyll fluorescence measurements are sensitive to excitation intensity and optical configuration, which can affect the measured fluorescence response [39].

### 4.4. Embedded Implementation and Practical Implications

One of the main contributions of this work is the deployment of the trained model on an embedded platform. The comparison between the Python model and its implementation on the ESP32 showed close numerical agreement, with only minor differences between implementations.

This result is relevant because the practical value of the system depends not only on model accuracy, but also on preserving the numerical behavior of the model after deployment. Under these conditions, the system is able to perform on-device inference without requiring external processing, which is particularly important for portable prepared-sample analysis.

The integration of sensing, processing, and communication within a single portable system represents an advantage over approaches that rely on laboratory analysis, external software, or cloud-based computation. Similar efforts have explored compact or energy-efficient models for environmental monitoring applications, highlighting the importance of embedded implementations and on-device inference in real-world scenarios [18,19]. In this case, the proposed system shows that multispectral fluorescence measurements combined with embedded machine learning can be used for rapid Chl-a estimation after sample preparation.

### 4.5. Limitations and Future Work

Despite the results obtained, some limitations should be considered. The model-development dataset covered 115.42–1809.13 µg/L, whereas the independent validation dataset covered only 203.74–934.94 µg/L. Therefore, the reported validation performance should be limited to this independently validated concentration interval. The lower and upper extremes of the model-development range, particularly concentrations above 934.94 µg/L, were not independently confirmed. Expanding the dataset, including additional samples at underrepresented concentration levels and the use of standard Chl-a solutions, would help improve the robustness and balance of the model.

In addition, the relatively low sample-to-feature ratio, approximately 3.2 for the model-development dataset, may increase the risk of overfitting and reduce the stability of feature-dependent patterns, particularly for nonlinear models. Although internal holdout testing, cross-validation, constrained model selection, and independent validation were used to reduce this risk, these strategies cannot fully eliminate the possibility that part of the learned relationship reflects variance induced by the limited sample size. Therefore, larger and more diverse datasets are required to better distinguish generalizable spectral relationships from spurious variance.

The model-development dataset was acquired using an earlier prototype version, whereas the independent validation dataset was acquired using the final hardware version. The main hardware changes were related to the transition from a perforated-board implementation to a PCB-based implementation and to the control of the excitation LEDs, while the dark chamber, optical geometry, cuvette position, and multispectral sensors were maintained. Although this configuration allowed a preliminary evaluation of model transfer between closely related hardware versions, it does not replace a complete calibration or recalibration using data acquired exclusively with the final device. The learned regression function may still depend on hardware-specific acquisition conditions, particularly excitation intensity and LED control. Therefore, the positive validation bias observed in this study may be partially associated with hardware-transfer effects. Future work should generate a larger calibration and validation dataset using only the final hardware version and should incorporate normalization or excitation-intensity correction strategies to improve model transferability and metrological traceability.

Further characterization of the system was not included in this work. A formal characterization using a stable fluorescent standard was not performed; therefore, future work should include fluorescent standard measurements to evaluate excitation stability, detector response stability, channel saturation margins, and the linear response range of the optical system. Although the raw digital outputs were inspected and no channel reached the 16-bit saturation limit in the dataset used for model development and independent validation, this analysis does not replace a complete optical characterization. In addition, a complete propagation of measurement uncertainty from the spectrophotometric reference method to the final Chl-a concentration was not performed. This analysis would require quantified uncertainty contributions from the main analytical steps, for example, filtered sample volume, extraction volume, solvent blank, absorbance measurements, cuvette path length, wavelength accuracy, and the calculation model used to obtain the final Chl-a concentration. Therefore, the residual-based U_res_ (*k* = 2) value reported in the validation table should be interpreted as an indicator of prediction residual dispersion rather than as a complete analytical or metrological uncertainty budget. Future work should include a full uncertainty budget for both the reference method and the portable fluorometric workflow.

Environmental acetone extracts may contain co-extracted pigments, degradation products, and fluorescent organic compounds that can contribute to the optical response. The spectrophotometric reference method used in this study was based on the trichromatic equation of Jeffrey and Humphrey, which partially accounts for spectral overlap among chlorophyll pigments by using absorbance measurements at multiple wavelengths. In addition, the 90% acetone blank correction accounted for the solvent background contribution, but it did not remove potential contributions from co-extracted compounds. Nevertheless, the selectivity of the proposed fluorometric model against individual interferents, such as pheophytin, carotenoids, or fluorescent dissolved organic matter, was not independently evaluated. Therefore, model performance should be interpreted within the matrix variability represented by the environmental acetone extracts analyzed in this study. Future work should include controlled mixtures and isolated interferents to further assess selectivity.

The present model was developed specifically for extracts prepared with 90% acetone. Therefore, direct application to extracts prepared with other solvents, such as methanol, ethanol, or chloroform, should not be assumed without additional calibration and validation. Solvent choice can influence pigment extraction efficiency, fluorescence yield, background signal, and spectral shape, which may modify the multispectral response used by the regression model. In addition, spectrophotometric equations and coefficients used for reference Chl-a quantification can be solvent-dependent. Accordingly, solvent-specific reference calculations, calibration, and validation would be required before applying the proposed workflow to other extraction solvents.

Finally, extending the methodology to raw water samples remains an open challenge. This would require a separate calibration and validation approach, since turbidity, suspended particles, temperature, and other matrix effects can influence the optical response [8,16,40]. The multispectral configuration may provide additional information to address these effects, but this application should be evaluated independently from the extracted-sample workflow presented in this study.

## 5. Conclusions

This work presented the development and evaluation of a portable system for estimating Chl-a concentration in acetone-extracted samples. The system integrates an optical fluorescence measurement stage, multispectral data acquisition, an embedded-compatible regression model, and an Android application for device control and result visualization. The validation results showed that the measured multispectral response can be related to Chl-a concentration within the independently validated concentration interval of 203.74–934.94 µg/L.

The integrated workflow, from optical measurement to on-device inference, was experimentally evaluated. The embedded implementation reduced the need for external processing hardware and preserved the numerical behavior of the original Python model. This is relevant because the system provides the estimated Chl-a concentration directly on the device, using only a mobile phone as the external interface.

Overall, the study demonstrates the feasibility of integrating multispectral fluorescence acquisition, embedded regression inference, local data storage, and mobile-device visualization into a portable system for Chl-a estimation in prepared acetone extracts. In practice, the proposed device may serve as a portable alternative for Chl-a estimation in prepared acetone extracts under the evaluated conditions. Once the extraction step is completed, the workflow is simplified through automatic acquisition, embedded concentration estimation, and local data storage. This may be useful in situations where access to conventional spectrophotometric or benchtop fluorometric equipment is limited.

Future developments will focus on improving model robustness, reducing the observed bias through recalibration or normalization, and expanding the dataset across underrepresented concentration ranges, especially below 203.74 µg/L and above 934.94 µg/L. The feasibility of extending the system to raw water samples may also be explored. However, this would require a separate calibration and validation approach.

## 6. Patents

This work is related to Mexican Patent Application No. MX/a/2025/015407, entitled “Dispositivo portátil híbrido para la determinación de clorofila-a mediante análisis fluorométrico multicanal y modelos embebidos de aprendizaje automático”, filed with the Instituto Mexicano de la Propiedad Industrial (IMPI; Mexican Institute of Industrial Property).

## Figures and Tables

**Table 1:** Nominal spectral characteristics of the AS7341 and AS7343 multispectral detector channels used as predictors in the regression model. Center/peak wavelengths and full width at half maximum (FWHM) values correspond to manufacturer-reported nominal or typical specifications. Clear, VIS, and FD correspond to broadband or auxiliary outputs without a defined narrowband center wavelength or FWHM.

Sensor	Channel	Center/Peak Wavelength [nm]	FWHM [nm]	Channel Type
AS7341	F1	415	26	Filtered spectral channel
AS7341	F2	445	30	Filtered spectral channel
AS7341	F3	480	36	Filtered spectral channel
AS7341	F4	515	39	Filtered spectral channel
AS7341	F5	555	39	Filtered spectral channel
AS7341	F6	590	40	Filtered spectral channel
AS7341	F7	630	50	Filtered spectral channel
AS7341	F8	680	52	Filtered spectral channel
AS7341	NIR	910	N/A	Near-infrared channel
AS7341	Clear	Si response/non-filtered	N/A	Broadband clear channel
AS7343	F1	405	30	Filtered spectral channel
AS7343	F2	425	22	Filtered spectral channel
AS7343	FZ	450	55	Filtered spectral channel
AS7343	F3	475	30	Filtered spectral channel
AS7343	F4	515	40	Filtered spectral channel
AS7343	FY	555	100	Filtered spectral channel
AS7343	F5	550	35	Filtered spectral channel
AS7343	FXL	600	80	Filtered spectral channel
AS7343	F6	640	50	Filtered spectral channel
AS7343	F7	690	55	Filtered spectral channel
AS7343	F8	745	60	Filtered spectral channel
AS7343	NIR	855	54	Near-infrared channel
AS7343	VIS	Si response/broadband visible	N/A	Broadband visible channel
AS7343	FD	Si response/non-filtered	N/A	Flicker detection/auxiliary channel

**Table 2:** Statistical summary of the chlorophyll-a (Chl-a) concentrations used in the model-development dataset (*n* = 76), including minimum, maximum, mean, standard deviation (SD), coefficient of variation (CV), median, quartiles, interquartile range (IQR), and skewness.

*n*	Minimum (µg/L)	Maximum (µg/L)	Range (µg/L)	Mean (µg/L)	SD (µg/L)	CV (%)	Median (µg/L)	Q1 (µg/L)	Q3 (µg/L)	IQR (µg/L)	Skewness
76	115.42	1809.13	1693.71	600.42	489.97	81.60	336.05	252.76	847.40	594.64	1.10

**Table 3:** Performance comparison of the evaluated machine learning models using R^2^, MAE, RMSE, and MAPE. The RF (TPOT-selected) row corresponds to the Random Forest model selected through the restricted TPOT optimization.

Model	*R* ^2^	MAE (µg/L)	RMSE (µg/L)	MAPE (%)
Ridge	0.992	26.37	37.17	4.57
Lasso	0.994	23.61	32.99	3.90
GBM	0.991	27.02	40.20	4.39
RFR	0.990	26.97	41.94	4.17
SVR	0.981	40.44	57.54	8.11
RF (TPOT-selected)	0.991	24.38	38.99	4.06

**Table 4:** Performance metrics of the model for the independent validation dataset, including *R*^2^, RMSE, MAE, bias, MAPE, RPD, and residual statistics. The reported residual-based U_res_ (*k* = 2) value was calculated from the standard deviation of the model residuals and summarizes the expanded residual dispersion of the model predictions.

*n*	*R* ^2^	RMSE (µg/L)	MAE (µg/L)	Bias (µg/L)	MAPE (%)	SD Ref (µg/L)	RPD	SD Residuals (µg/L)	U_res_ (*k* = 2) (µg/L)
15	0.979	35.41	28.91	22.00	6.14	255.06	7.20	28.72	57.43

**Table 5:** Repeatability and drift analysis for three representative chlorophyll-a (Chl-a) concentrations. The table includes mean, standard deviation (SD), coefficient of variation (CV), repeatability limit (r), and drift parameters obtained from repeated measurements.

Concentration (µg/L)	*n*	Mean (µg/L)	SD (µg/L)	CV (%)	Repeatability Limit, *r* (µg/L)	Drift Slope (µg/L per Measurement)	Estimated Total Drift (µg/L)	*R*^2^ (Drift)	*p*-Value (Drift)
934.92	10	981.31	32.30	3.29	90.43	−5.72	−51.45	0.29	0.11
558.37	10	530.59	12.47	2.35	34.91	−2.65	−23.83	0.41	0.04
229.06	10	235.37	0.91	0.39	2.55	0.11	1.01	0.14	0.29

**Table 6:** Comparison between the predictions obtained from the Python model and those generated by the embedded implementation on the ESP32. The table includes error metrics (MAE, RMSE, and bias), maximum absolute difference, and the coefficient of determination (*R*^2^) used to evaluate consistency between both implementations.

*n*	MAE (µg/L)	RMSE (µg/L)	Bias (µg/L)	Max Absolute Difference (µg/L)	*R* ^2^
15	10.75	14.81	−0.07	35.63	0.997

## Data Availability

The data presented in this study are available on request from the corresponding author.
